# Local Anæsthesia

**Published:** 1855-02

**Authors:** 


*Local Anæsthesia.*—A writer in one of our exchanges, not long since, gave an amusing account of an attempt to produce local anæsthesia by the topical application of chloroform. The smarting from the chloroform was so much worse than the pain of the operation would have been, that his patient left him in high dudgeon, and cursing vehemently. We laughed the harder at the incident from a very recent memory of a similar attempt on our own part, which resulted in inglorious failure. We advise all who wish to preserve their *amour propré*, to avoid the topical application of chloroform as a preparation to surgical operations.

Anæsthesia by cold is, however, a different matter. If a nerve is well frozen, we will answer for it that it will not flinch at the knife. A bag of pounded ice and salt applied to the part will soon benumb it past sensation, and so far as yet known, does not interfere with the subsequent union of incisions.

This reminds us to ask what has become of the use of cold in erysipelas. A number of cases were formerly reported in this Journal when the chilling of the inflamed surface by the action of the bag of salt and ice resulted very favorably. In fact, we have never heard of any bad results from it, but it seems to have gone out of use without any sufficient cause. We do not believe that so sudden a check to the inflammation can be prudent in the early stages, but when the disease is far advanced, the inflammation excessive, and as we think a natural consequence, the elimination of the specific poison nearly or quite complete, then there can be little doubt as to its propriety. It must control the local action, which is then really all that remains of the disease.

From the topical application of chloroform to the treatment of erysipelas how easy the grade! H.

